# Collectivism, face concern and Chinese-style lurking among university students: the moderating role of trait mindfulness

**DOI:** 10.3389/fpsyg.2024.1298357

**Published:** 2024-02-21

**Authors:** Bing Hu, Yi Zhu, Chao Liu, Shanshan Zheng, Ziying Zhao, Ruxiang Bao

**Affiliations:** ^1^School of Journalism and Communication, Huaqiao University, Xiamen, China; ^2^School of Economics and Finance, Huaqiao University, Quanzhou, China; ^3^Business Analytics Research Center, Chang Gung University, Taoyuan, Taiwan

**Keywords:** Chinese-style lurking, collectivism, face concern, online social anxiety, trait mindfulness

## Abstract

**Introduction:**

This study focuses on understanding the unique causes and mechanisms of “Chinese-style lurking” on WeChat among university students, within a cultural context that emphasizes collectivism and face concern. The research also looks into the moderating role of trait mindfulness.

**Methods:**

For the confirmation of these phenomena and to validate the theories, a structural equation model was constructed using the Stress-Strain-Outcome (SSO) theory and mindfulness buffering theory. The model was then tested and validated with data from 1,453 valid online surveys. These data were analyzed using the SmartPLS 4.0 software.

**Results:**

The results indicate that collectivism increases face concern, which in turn escalates online social anxiety. Face concern completely mediates between collectivism and online social anxiety, creating a serial mediation effect between face concern, online social anxiety, and lurking behavior. Additionally, trait mindfulness was found to negatively modulate the pathways from collectivism to face concern and from online social anxiety to lurking.

**Discussion:**

The findings underscore the influence of traditional Chinese culture on contemporary students' online behavior and provide a new perspective for understanding social media lurking in an Eastern context. The results suggest that a mindfulness-based approach could be used to mitigate the associated silence and anxiety.

## 1 Introduction

The United Nations (2014) underscores the significance of citizen involvement in social development. Moreover, both the World Programme of Action for Youth and the United Nations (n.d.) affirm the pivotal role of young people as catalysts for innovation and change, emphasizing that their active engagement in policy processes and expression of opinions are crucial for promoting inclusive and participatory governance, ultimately contributing to the establishment of sustainable and stable societies. In the context of China, WeChat serves as a vital platform for young “digital natives” to express their opinions and participate in societal matters. However, in recent years, an increasing number of Chinese university students have begun hiding or withdrawing from the lively social functions of WeChat, adopting a unique “Chinese-style lurking” posture to enter the research perspective of media and health communication.

The term “Chinese-style lurking” pertains to the social media conduct modulated by indigenous Chinese cultural traditions and ideologies such as Confucianism, collectivism, and face-saving tendency. These norms are assimilated into users' self-identity, engendering distinct traits of online social anxiety–encompassing fear for evaluation, privacy concern, and social anxiety– within their digital interactions. This culminates in a behavioral pattern on social platforms wherein users maintain presence yet abstain from active engagement like commenting or voicing personal opinions. “Chinese-style lurking” is more than just an extension and perpetuation of the “most nuanced standards of Chinese social exchanges” (Zhang and Wang, 2019); it symbolizes the fusion and interplay of traditional Chinese culture with modern information and media culture. Above all, it emerges as a pivotal ground for distinguishing Eastern and Western cultures and for elucidating individual attitudes and actions (Arpaci, 2014). This concept differs markedly from the Western concept of “lurking” prevalent in previous studies (Ortiz et al., 2018). Western studies on lurking have explored factors such as individuals, society, technology, and organizations, including computer anxiety and privacy risks (Soroka and Rafaeli, 2006; Nguyen, 2021). In contrast, the concept of “Chinese-style lurking” within the “person-culture fit” framework (Oishi et al., 1999) demonstrates more influences on uncertain aspects, particularly in relation to collectivism (De Vaate et al., 2020). The concept of “face,” closely linked to collectivism in Chinese culture, subtly shapes the psychology and behavior of individuals. Our study aims to investigate the mechanism of “Chinese-style lurking” among university students within the cultural framework of collectivism and face concern. By constructing a structural equation model that incorporates the Social Support Online (SSO) model and mindful stress buffering theory, the research seeks to identify the unique factors and underlying mechanisms driving this behavior. Additionally, the moderating role of trait mindfulness in this process will be explored. The findings will enhance our understanding of the psychological and sociocultural factors that shape online behavior among Chinese youth.

## 2 Theoretical background and research hypothesis

### 2.1 Theoretical background

#### 2.1.1 Theory of stress-strain-outcome

The SSO Theory, originating from Koeske and Crouse (1981) study on occupational burnout, is a widely recognized and extensively used model in social psychology. It examines the impact of external pressures on individual psychological and behavioral outcomes. The SSO framework consists of stressors, strains, and outcomes, with strains playing a mediating role between stressors and behavioral outcomes (Koeske and Koeske, 1993), which has been successfully applied in various domains of health communication, including emotional disorders (Cheung and Tang, 2010), health information sharing (Wang and Zhu, 2020), alcohol addiction (Henning, 1986), psychological wellbeing (Dhir et al., 2018), and social media fatigue (Dhir et al., 2019). Considering that lurking behavior is a typical manifestation of social media burnout (Liu et al., 2017), this study introduces the SSO theoretical framework to explain the Chinese-style lurking behavior.

Stressors refer to objective events in the environment that individuals perceive as bothersome or potentially destructive. In collectivist cultures, sacrifice and submission are prevalent traits (Park et al., 2005). Coupled with the influence of face concern, individuals may experience stress and anxiety in order to preserve and enhance their face. When using WeChat Moments, Chinese youth tend to be highly sensitive to evaluations from others within their social circle (Wang and Lan, 2017). This sensitivity aims to achieve harmony and maintain their own reputation, but it also leads to anxiety when expressing themselves online (Song, 2022) and ultimately results in heightened levels of social anxiety (Liang and Duan, 2018). Therefore, collectivism and face concern can be regarded as important stressors leading to individual distress and anxiety.

Strains refer to the destructive negative adaptation in various aspects, such as attention, psychology, and emotions, that individuals experience as a result of being influenced by stressors. Online social anxiety can result in psychological exhaustion (Liu and Ma, 2020), negative social media usage (Hong et al., 2015), and other negative consequences. Individuals often resort to negative coping mechanisms such as seeking self-comfort, relying on others, indulging in fantasy, and engaging in deliberate forgetting to evade problems and alleviate anxiety. However, this pattern of “anxiety situation → avoidance → anxiety relief” only traps individuals in an endless cycle of social anxiety (Li and Qian, 2003). Therefore, online social anxiety can be seen as a form of strain that individuals develop in response to stressors, subsequently leading to a cascade of negative psychological and behavioral responses.

Outcomes refer to the enduring sense of stress or tension that individuals experience when exposed to stressors, resulting in persistent negative behaviors or psychological reactions. Nonnecke et al. (2004) reveal that prolonged anxiety can induce psychological changes in young individuals. As a result, they develop a belief that maintaining silence online is a means of ensuring their safety. Consequently, this perception further intensifies the prevalence of lurking behavior. Lurking behavior can be conceptualized as a typical outcome demonstrated by social media users, which is influenced by multiple factors such as anxiety (Liu et al., 2018), role stress (Guo, 2016), and privacy concerns (Wan and Cheng, 2015; Zhu, 2020).

#### 2.1.2 Mindfulness stress–buffering theory

The mindful stress buffering theory provides a theoretical approach to the relationship between mindfulness and health outcomes, proposing that mindfulness reduces stress assessment as well as responses to stress, and that these stress-relieving effects partially or fully explain how mindfulness affects mental and physical health (Creswell, 2014; Voss et al., 2020). Mindfulness is closely related to emotional, psychological, social, and overall health (Howell et al., 2008), and high mindfulness not only corresponds to lower levels of symptoms of depression, anxiety, and stress (Siegling and Petrides, 2014; Bravo et al., 2017; Valikhani et al., 2018), but at the same time, it also enables users to be less affected by negative feedback on social media (Valkenburg et al., 2006; Liu et al., 2023). Therefore, the theoretical framework of this study is built on the integration of the above two theories. Based on the SSO model, collectivism and face concern are regarded as the sources of stress affecting Chinese college students' social media use, and the moderating effect of trait mindfulness on the above stress is investigated.

### 2.2 Research hypothesis

#### 2.2.1 Collectivism and face concern

Face concern encompasses the recognition and aspiration of individuals to enhance, preserve, and avoid losing face in their interactions with others (Bao et al., 2003). Informed by the principles of Confucianism, China has nurtured a deep-rooted cultural tradition of “saving face” (Kwek et al., 2019; Gürel et al., 2021; Wang and Tian, 2022). In Chinese culture, face represents not only an individual's own prestige but also the reputation of broader social groups, including family, relatives, friends, and even colleagues (Hwang, 1987). Face concern not only exhibits a salient feature of Chinese social behavior (Cui and Mei, 2021) but also constitutes a cultural cornerstone of Asian collectivism (Chan et al., 2009). Furthermore, collectivism accentuates the notion of “interdependence among individuals within a group” (Sorensen, 2011; Msetfi et al., 2015). In collectivist societies, the individual's face exerts a substantial impact on the group's reputation (Lee and Sparks, 2007; Qi, 2011), while also fostering in-group preference and a multitude of face preservation strategies in interpersonal relationships (Oyserman et al., 2002). Thus, from this point, collectivism reinforces the face concern among Chinese individuals. Therefore, the following hypothesis is proposed.

H1: Collectivism has a positive effect on face concern.

#### 2.2.2 Online social anxiety and lurking

Online social anxiety can be viewed as an extension of offline social anxiety in the context of virtual interactions, characterized by an irrational fear of being scrutinized or evaluated by others in social situations (Morrison and Heimberg, 2013). The cognitive behavioral model of social anxiety (Lu et al., 2019) suggests that individuals who have strong desire to uphold their reputation (Zhang, 2011) tend to engage in active and responsible behaviors within their social circles to cultivate a favorable self-image. However, when confronted with situations that could lead to face loss, such as receiving aggressive or hostile responses or observing mistreatment from other group members, worrying about information being disregarded or damaging one's own and the collective reputation, individuals are prone to experiencing excessive fear of potential embarrassment or humiliation known as social anxiety (Liu and Cheng, 2023). Previous studies have noted the similarities between online and offline manifestations of social anxiety (Weidman and Levinson, 2015). For example, platforms such as Facebook (Sindermann et al., 2020) and Instagram exhibit clear anxieties and fears associated with negative evaluations, which are not limited to either online or offline contexts (Jiang and Ngien, 2020). Based on these observations, we propose the following hypotheses.

H2: Collectivism positively affects online social anxiety.
H3: Face concern positively affects online social anxiety.

Lurking behavior refers to the act of social media users observing activities on social media platforms without actively participating (Ortiz et al., 2018). Due to the awareness of risk aversion to avoid losing face (Belk, 1988) and the need to alleviate the pressure and tension arising from online social anxiety (Zhang et al., 2021), an increasing number of Chinese youth, including WeChat users, opt for conservative and cautious lurking behaviors (Dhir et al., 2018; Hong et al., 2023). This perpetual cycle of silent diving and social anxiety can lead users to develop a negative attitude toward social media and diminish their intention to continue using it, ultimately resulting in a propensity for diving (Weidman and Levinson, 2015). Based on this, the following hypothesis is proposed.

H4: Face concern positively affects lurking behavior.
H5: Online social anxiety positively affects lurking behavior.

With regard to the analysis of hypotheses H1, H2, and H3, we infer that face concern plays a mediating role between collectivism and online social anxiety. Additionally, hypotheses H4 and H5 propose that online social anxiety acts as a mediator between face concern and social media diving behavior. Moreover, compared to the single mediation model, the multi-mediation model includes both single mediation and serial mediation paths, which provides a more comprehensive understanding of the interaction mechanism between variables. Within the context of collectivism, face concern can stimulate online social anxiety, thereby intensifying lurking behavior prevalence. Therefore, both face concern and online social anxiety can act as a single mediator as well as serial multiple mediators. We propose the following hypotheses.

H6: Face concern mediates the relationship between collectivism and online social anxiety.
H7: Online social anxiety mediates the relationship between face concern and lurking behavior.
H8: Face concern and online social anxiety act as serial multiple mediators between collectivism and lurking behavior.

#### 2.2.3 The moderating role of trait mindfulness

The moderating role of mindfulness between collectivism and face concern has not been sufficiently addressed in previous research. Individuals with collectivist orientation tend to prioritize others and engage in other-oriented or mutual-oriented attention preferences (Ting-Toomey and Kurogi, 1998; Liu et al., 2020a,b). They engage in acts of service and sacrifice (Singelis et al., 1995) to gain respect and recognition from others within the collective and enhance their face. As a result, individuals' self-perception and emotional experiences in collectivist and face concern contexts often exhibit an external focus, preconceived notions, and a lack of self-relevance. On the other hand, mindfulness is defined as non-judgmental and accepting attention that is focused on present-moment activities, as well as internal thoughts and feelings (Bishop et al., 2004). Through the influence of trait mindfulness, individuals can consciously transfer the stress control point from the external conditions of the collective situation to the internal metacognition and attention resources (Van Gordon et al., 2014). By focusing on the task at hand and accurately acknowledging their own need for face, they can effectively alleviate the face pressure associated with collectivism (Ghauri and Fang, 2001; Lee and Sparks, 2007). Accordingly, the following hypotheses are proposed.

H9: Trait mindfulness negatively moderates the relationship between collectivism and face concern.

Recent research has demonstrated that young individuals experience a significant increase in negative emotions when they receive negative or unexpected feedback on social media, such as not receiving the expected number of likes (Jiang, 2013). This heightened negative emotional state contributes to online social anxiety and engagement in lurking behavior (Dhir et al., 2018). Moreover, trait mindfulness has emerged as a potential regulator in this process, empowering individuals to effectively rectify distorted self-perceptions (Crescentini and Capurso, 2015) and readjust their understanding of personal identity and relationships with others or collectives. Consequently, it reduces individuals' fear of negative online evaluation and enhances their ability to regulate negative emotions (Gámez-Guadix and Calvete, 2016; Guendelman et al., 2017), thus reducing lurking behavior triggered by online social anxiety. Based on this, we propose the following hypothesis.

H10: Trait mindfulness negatively moderates the relationship between online social anxiety and lurking behavior.

**Figure 1:**
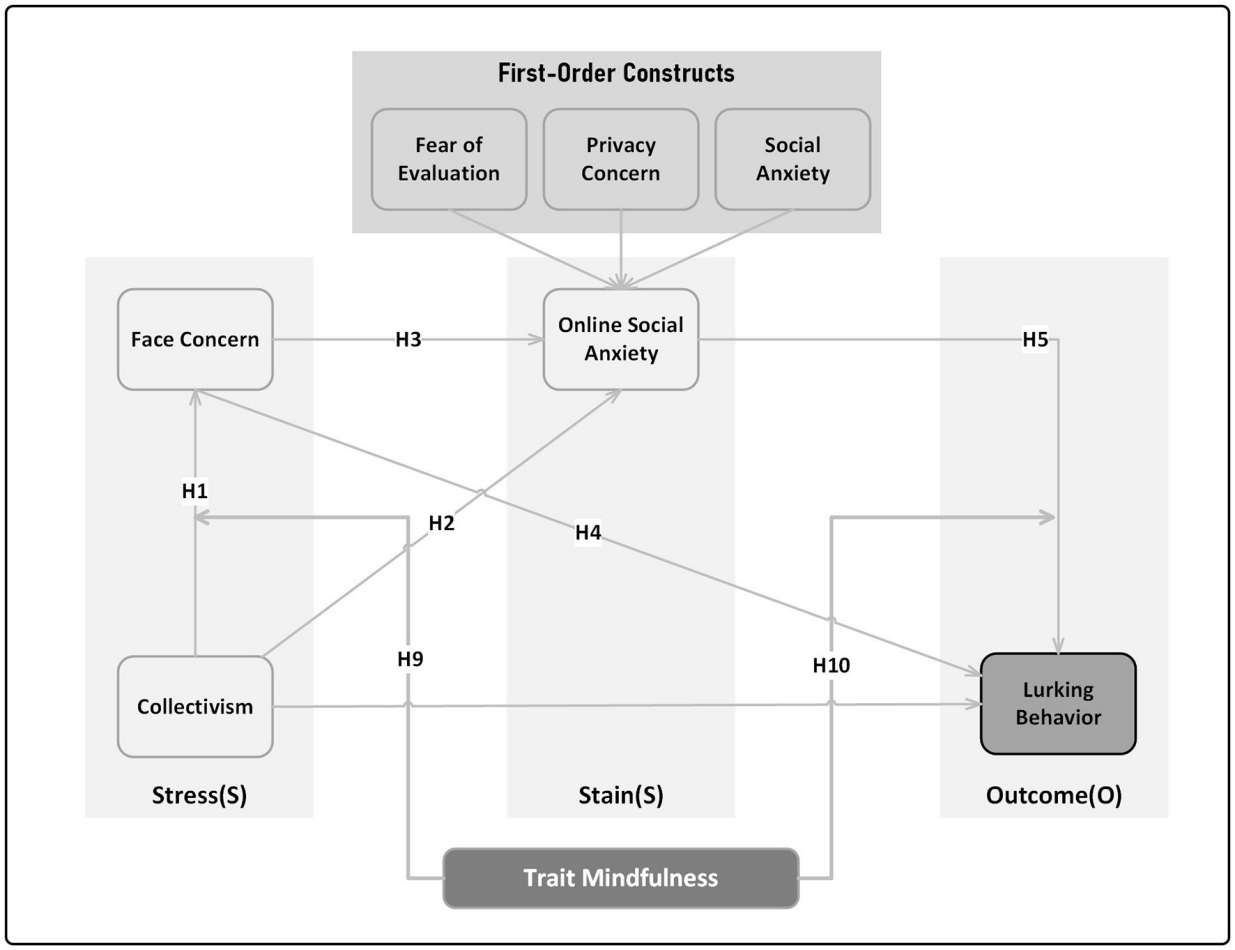
Conceptual framework and research hypotheses.

Based on the aforementioned, this study employs the SSO theoretical framework, wherein face concern is regarded as a stressor rooted in collectivist cultures, online social anxiety serves as the strain, and lurking behavior represents the outcome, delving into the underlying mechanism of lurking behavior among Chinese university students. Moreover, it explores the moderating role of trait mindfulness. The conceptual framework is visually depicted in Figure 1.

## 3 Method

### 3.1 Participants and data collection

Our study focuses on Chinese university students, aiming to gather a comprehensive sample that covers variations in geographical location, university type, grade, and major. Considering budget constraints, feasibility, and sample accessibility, online sampling was selected as the most practical approach. However, this method has inherent selection biases, potentially leading to an overrepresentation or underrepresentation of certain groups. For instance, students from specific provinces might have more active participation or easier access to the survey, which could introduce regional biases. Such biases are particularly concerning as they can affect the accuracy of SEM analyses and limit the generalizability of the study's findings. To address these issues, we employs a dual strategy of stratified and online sampling, facilitated by Questionnaire Star. This leading Chinese online survey platform boasts a database of over 10 million high-quality, demographically diverse samples. Its advanced screening capabilities enable precise targeting of desired participant groups, ensuring a comprehensive and representative sample. Using Questionnaire Star's broad reach, a robust sampling framework was established, employing the platform's random distribution function to ensure equal selection chances for all potential participants. Stratification was based on geographical region, academic major, and year of study, capturing a wide representation of the Chinese university student population and bolstering sample diversity. The sample notably included respondents from diverse provinces such as Fujian, Zhejiang, Guangdong, Hunan, Jiangxi, Gansu, Jilin, and Heilongjiang, representing eastern, central, western, and northeastern China. To optimize the sampling process, we took two supplementary measures: First, we fine-tuned the questionnaire, aiming at elevated comprehension and engagement. Second, we expanded our survey reach through various promotional mediums, including personal networks, to access an extensive respondent base and foster diversity within the sample.

To meet the correlation coefficient requirements, the required sample size was estimated using G^*^Power 3.1.9.7 statistical analysis software, with one-way ANOVA and *t* test between to independent means used. For the first calculation, one-way ANOVA was chosen with settings of five groups, an effect value of 0.25, statistical test power 1-β 0.95, and a probability of one-class error a of 0.05, resulting in a minimum total sample size of 305. For the second calculation, *t*-test between to independent means was chosen with settings of the same effect values, statistical test power, and the allocation ratio set to 1, resulting in a minimum total sample size of 834. Therefore, the minimum sample size required for this survey was concluded to be 834.

In the first stage of date collection, a preliminary survey was conducted on June 20, 2023. A total of 111 questionnaires were recovered from the Questionnaire Star platform, and 95 were valid. The questionnaires were adjusted and modified according to the reliability and validity evaluation results. The second phase of the formal survey was conducted, and questionnaires were distributed and collected from July 22 to August 22, 2023. A total of 1,647 questionnaires were collected, 194 invalid questionnaires were excluded, and 1,453 valid questionnaires were obtained, with an effective response rate of 88.2%, which fulfilled the sample size requirement for analysis.

Table 1 presents the demographic characteristics of the sample. The majority of participants (80%) fell within the 18–24 age range. 72.3% of the participants are undergraduate students, while 22.7% are postgraduates. Liberal arts students comprised 54.8%, while science and engineering students comprised 45.2%. Furthermore, 63.7% of the participants used WeChat for 2–6 h daily, while 22.4% used it for over 6 h every day. Overall, the samples align with the required characteristics of the study.

**Table 1:** Demographic characteristics of participants (*n* = 1,453).

**Characteristics**	**Demographic information**	**Frequency**	**%**
Gender	Male	595	40.9
	Female	858	59.1
Grade	Freshman	214	14.7
	Sophomore	363	25.0
	Junior	316	21.7
	Senior (and fifth)^a^	158	10.9
	Master degree or above	402	27.7
Age	18 and below	54	3.7
	18–24	1,162	80.0
	25–30	215	14.8
	30 and above	22	1.5
Major	Liberal arts	796	54.8
	Science and engineering	657	45.2
Daily usage time of WeChat	Within 2 h	202	13.9
	2–4 h	481	33.1
	4–6 h	444	30.6
	6–8 h	197	13.6
	More than 8 h	129	8.9
Duration of WeChat usage	3 years and below	97	6.7
	3–5 years	540	37.2
	5–10 years	688	47.4
	More than 10 years	128	8.8
Family residence	Countryside	629	43.3
	Cities and towns	824	56.7

a Most majors in Chinese universities generally have a 4-year schooling system, and medical majors have a 5-year schooling system.

### 3.2 Measures

To ensure measurement validity and reliability, all questionnaire items were adapted from validated scales featured in prior academic publications. The scales for two critical variables— collectivism and face concern—were adapted and tested by Chinese scholars within a Chinese sample environment, effectively capturing the unique aspects of Chinese cultural consciousness. Considering that the survey was conducted amongst Chinese respondents, a bilingual translation approach was employed to culturally tailor the English scales. A process of forward and backward translation—English to Chinese, then Chinese back to English—was carried out by a doctoral researcher and an English-speaking graduate student.

Except for the trait mindfulness measurement which was evaluated on a 6-point scale, all other variables in this study were measured using Likert 5-point scale (1 = strongly disagree, 2 = disagree, 3 = neutral, 4 = agree, 5 = strongly agree). All scales employed in this study were obtained from validated measures documented in previously published academic papers. Considering that the survey was conducted in China and to accommodate the language and cultural background of the Chinese participants, a bilingual translation approach was employed. The scales were initially translated from English to Chinese by two doctoral researchers and three master's students proficient in English (forward translation). Then, the Chinese version was translated back into English (backward translation) to ensure the accuracy and clarity of the questionnaire presentation.

#### 3.2.1 Collectivism

Collectivism (abbreviated as CL) was measured employing the Chinese version of the Individualism-Collectivism Scale (ICSC) developed by Triandis and Gelfand (1998) and revised by Huang et al. (2014). The scale consisted of eight items, such as “Respecting the decisions of the group is extremely important to me,” and “If my colleague (classmate) receives recognition, I will be pleased for him.” The scale demonstrated good reliability with Cronbach's alpha = 0.838, M = 3.719, SD = 0.664.

#### 3.2.2 Face concern

To measure face concern (abbreviated as FC), we utilized the Face Concern Scale employed in the study conducted by Chan et al. (2009), which was adapted from the works of Cocroft and Ting-Toomey (1994) and White et al. (2004). This scale comprises six items, such as “I care about others' attitudes toward me” and “I feel highly satisfied when treated with respect.” Higher scores indicate a stronger level of face concern exhibited by individuals. The scale demonstrated good reliability with Cronbach's α = 0.857, M = 3.793, SD = 0.769.

#### 3.2.3 Online social anxiety

To measure online social anxiety (abbreviated as OSA), we utilized the Chinese version of the Social Anxiety Scale for Social Media Users (SAS-SMU) developed by Alkis et al. (2017) and revised by Chen et al. (2014). The scale consists of 22 items that are categorized into three dimensions: Fear of Evaluation (FE), Privacy Concerns (PC), and Social Anxiety (SA). Example items include “I worry that what I share will be mocked by others,” “I feel anxious that my private information might be shared publicly,” and “I feel nervous when interacting with unfamiliar individuals,” etc. The FE measurement items Cronbach's α = 0.912, M = 3.418, SD = 0.897; PC items Cronbach's α = 0.886, M = 3.600, SD = 0.895; SA measurement items Cronbach's α = 0.932, M = 3.272, SD = 0.918.

The SAS-SMU scale structure categorizes online social anxiety (OSA) as a second-order construct, whereas Fear of Evaluation (FE), Privacy Concerns (PC), and Social Anxiety (SA) are classified as first-order measurements. According to the decision rules proposed by Jarvis et al. (2003) for conceptual measurement models,^1^ the relationship between OSA and its subordinate first-order measurements exhibits a formative relationship. This classification falls under the Reflective-Formative Higher-Order construct defined by Hair et al. (2011).

#### 3.2.4 Lurking behavior

To measure Lurking Behavior (abbreviated as LB), the study employed the Online Lurkers scale developed by Nonnecke et al. (2004) and made appropriate adaptations to fit the usage patterns of Chinese WeChat users. The scale consists of four items, including “Sometimes I just lurk in WeChat groups, reading others' messages without responding” and “I often just browse through updates and statuses in WeChat Moments without reacting, rarely liking or commenting on them.” The scale demonstrated good reliability with Cronbach's α = 0.805, M = 3.375, SD = 0.924.

#### 3.2.5 Trait mindfulness

To measure participants' trait mindfulness levels, the study employed the Mindful Attention Awareness Scale (MAAS) developed by Brown and Ryan (2003). This unidimensional scale comprises 15 items, rated on a response scale ranging from “1” (almost always) to “6” (almost never). Higher scores on the MAAS indicate a greater propensity for individuals to exhibit heightened awareness and attention to the present moment in their daily lives. The scale demonstrated good reliability with Cronbach's α = 0.932, M = 4.098, SD = 0.915.

### 3.3 Data analysis methods

Multivariate normality test conducted with Stata 15.0 revealed that the sample data did not conform to multivariate normal distribution (Mardia's Multivariate Kurtosis = 2,223.892, *P* < 0.001; Mardia's Multivariate Skewness = 147.654, *P* < 0.001). Despite such divergence, the PLS-based SEM modeling exhibits robustness when applied to highly skewed data (Cassel et al., 1999; Reinartz et al., 2009). Furthermore, our study's key variable OSA is recognized as a Reflective-Formative second-order construct. Given CB-SEM algorithms' limitations in handling such constructs, our modeling was carried out using SmartPLS 4.0 which employs a more adaptable PLS-SEM approach. Descriptive statistical analysis, independent samples *t*-test, reliability analysis, one-way analysis of variance (ANOVA), Pearson correlation analysis, and exploratory factor analysis were performed using SPSS25.0. SmartPLS 4.0 was employed to assess the construct's reliability and validity, conduct structural equation modeling (SEM), and evaluate model fit. The indicators used in the analysis included path coefficients (β), R2, SRMR, Chi^2^, and NFI.

To estimate the Reflective-Formative Second-order construct, we utilized the disjoint two-stage approach as recommended by Sarstedt et al. (2019). In the first stage, we estimated all other constructs in the path model using their standard multi-item measures. Subsequently, we saved the construct scores that only included the lower-order components. In the second stage, these saved first-order latent variable scores were employed as measurement indicators of the higher-order construct, thus facilitating the successful fitting of the SEM model.

## 4 Data analysis results

### 4.1 Differential test of demographic characteristics

Demographic differences in social media lurking behavior among Chinese university students were examined using SPSS 25 software through *t*-tests and ANOVA tests. The findings indicated significant variations in social media lurking behavior, which were influenced by two variables: grade and age (*p* < 0.05). Notably, significant differences were found in Freshman university students, individuals aged 25–30, and those above 30 years old. However, there were no significant differences observed among different genders, majors, and family residence, as indicated in Table 2.

**Table 2:** Differential testing of demographic characteristics in lurking behavior (*n* = 1,453).

**Characteristics**	**Demographic information**	**Mean**	**SD**	**T or F**	***p*-value**	**Multiple comparisons**
Gender	Male	3.385	0.971	0.316	0.270	
	Female	3.369	0.891			
Grade	Freshman	3.102	1.029	7.718	0.000	Freshman
	Sophomore	3.325	0.917			
	Junior	3.529	0.876			
	Senior (and fifth)	3.442	0.904			
	Master degree or above	3.419	0.885			
Age	18 and below	3.208	0.965	9.577	0.000	25–30, 30 and above
	18–24	3.358	0.921			
	25–30	3.590	0.877			
	30 and above	2.625	0.928			
Major	Liberal arts	3.385	0.894	0.542	0.588	
	Science and engineering	3.357	0.979			
Family residence	Countryside	3.393	0.954	0.628	0.530	
	Cities and towns	3.362	0.902			

### 4.2 Reliability and validity testing

Table 3 presents the cross-loadings among latent variable indicators, revealing that the internal loadings of items measuring all reflective constructs are significantly higher than their cross-loadings on other constructs, thereby demonstrating discriminant validity. The regression weights between the second-order formative latent variable, OSA, and its subordinate first-order latent variable scores (FE^*^, PC^*^, SA^*^) are 0.476, 0.302, and 0.222, surpassing the threshold of 0.2 (Diamantopoulos, 2006) and achieving statistical significance at the 1% level. This indicates the presence of item reliability in the formative measurement. Furthermore, the VIF of the above three first-order latent variables are all below 3.3, meeting the collinearity requirement for formative measurement (Kock and Lynn, 2012).

**Table 3:** Reliability, convergent validity, and cross-loadings/weights.

**Variable**	**Form**	**Item**	**FC**	**CL**	**LB**	**OSA**	**TM**	**α**	**VIF**	**AVE/t**
Face concern	Reflective	FC1	**0.801**	0.375	0.238	0.383	0.139	0.857		0.576
		FC2	**0.800**	0.407	0.201	0.418	0.126			
		FC3	**0.802**	0.414	0.222	0.394	0.112			
		FC4	**0.765**	0.451	0.199	0.349	0.174			
		FC5	**0.749**	0.557	0.192	0.284	0.225			
		FC6	**0.719**	0.395	0.192	0.393	0.152			
Collectivism	Reflective	CL1	0.448	**0.792**	0.111	0.186	0.243	0.838		0.440
		CL2	0.447	**0.791**	0.130	0.228	0.243			
		CL3	0.325	**0.575**	0.084	0.202	0.216			
		CL4	0.361	**0.639**	0.104	0.167	0.278			
		CL5	0.248	**0.539**	0.093	0.183	0.253			
		CL6	0.287	**0.508**	0.120	0.132	0.234			
		CL7	0.415	**0.734**	0.152	0.201	0.244			
		CL8	0.406	**0.719**	0.125	0.247	0.256			
Online social anxiety	Formative	FE^*^	0.474	0.269	0.407	**0.476**	0.322	0.912	2.893	8.275
		PC^*^	0.393	0.276	0.411	**0.302**	0.341	0.886	2.096	6.652
		SA^*^	0.328	0.201	0.455	**0.222**	0.253	0.932	2.250	4.004
Lurking behavior	Reflective	LB1	0.168	0.089	**0.724**	0.301	0.209	0.865		0.576
		LB2	0.106	0.086	**0.758**	0.285	0.194			
		LB3	0.212	0.179	**0.764**	0.343	0.232			
		LB4	0.290	0.140	**0.792**	0.451	0.155			
Trait mindfulness	Reflective	TM1	0.139	0.279	0.217	0.244	**0.713**	0.932		0.514
		TM2	0.141	0.245	0.208	0.234	**0.701**			
		TM3	0.158	0.245	0.176	0.256	**0.747**			
		TM4	0.149	0.243	0.125	0.280	**0.652**			
		TM5	0.150	0.246	0.178	0.262	**0.709**			
		TM6	0.167	0.245	0.159	0.279	**0.667**			
		TM7	0.150	0.236	0.178	0.256	**0.761**			
		TM8	0.146	0.271	0.166	0.219	**0.790**			
		TM9	0.093	0.202	0.201	0.256	**0.769**			
		TM10	0.148	0.245	0.167	0.255	**0.769**			
		TM11	0.100	0.228	0.162	0.264	**0.712**			
		TM12	0.200	0.252	0.215	0.276	**0.710**			
		TM13	0.030	0.162	0.156	0.203	**0.644**			
		TM14	0.089	0.193	0.175	0.196	**0.730**			
		TM15	0.250	0.316	0.215	0.273	**0.675**			

Bold values denote the internal loadings of items measuring their own constructs, and non-bold values indicate cross-loadings on other constructs. FE^*^, PC^*^, and SA^*^ are the latent variable scores obtained in the first stage of the two-stage approach (Sarstedt et al., 2019) for the reflective-formative second-order construct OSA. As formative indicators do not have loadings, composite reliability (CR), or average variance extracted (AVE), regression weights (weigh), t-values, and VIF are provided for FE^*^, PC^*^, and SA^*^. The threshold for weights is set at >0.2 (Chin, 1998). The α represents the Cronbach's α coefficient for the latent variable in the table.

Table 4 confirms the convergent validity of the study, with all latent variables exhibiting CR values above 0.7 and most first-order constructs surpassing the AVE threshold of 0.5. The Collectivism variable, while slightly below the AVE threshold at 0.440, still meets the criteria as its CR value exceeds 0.6, suggesting adequate convergent validity according to Fornell and Larcker (1981). Additionally, all intercorrelations among latent variables are lower than the square root of their respective AVEs, indicating good discriminant validity. Overall, these results demonstrate the strong validity of the measurement in this study.

**Table 4:** Correlation analysis and discriminant validity (Fornell and Larcker criterion).

	**AVE**	**CR**	**CL**	**FC**	**FE**	**PC**	**SA**	**TM**	**LB**
CL	0.440	0.893	**(0.663)**						
FC	0.576	0.927	0.641^***^	**(0.759)**					
FE	0.640	0.947	0.325^***^	0.566^***^	**(0.800)**				
PC	0.673	0.946	0.291^***^	0.471^***^	0.761^***^	**(0.820)**			
SA	0.623	0.954	0.225^***^	0.386^***^	0.754^***^	0.687^***^	**(0.789)**		
TM	0.514	0.948	0.382^***^	0.206^***^	0.336^***^	0.365^***^	0.263^***^	**(0.717)**	
LB	0.576	0.916	0.205^***^	0.318^***^	0.463^***^	0.489^***^	0.545^***^	0.304^***^	**(0.759)**

The bold values indicated the square root of AVE.

*** *p* < 0.001.

### 4.3 Common method bias

Data collected through questionnaire surveys may suffer from common method bias, to address this, the Harman's single-factor test was employed. The analysis resulted in the extraction of seven factors, each with eigenvalues >1. The first factor accounted for 28.039% of the total variance, which fell below the 40% threshold, indicating an acceptable level of common method bias. Additionally, following Kock (2015) perspective, VIF above 3.3 is considered indicative of pathological collinearity and potential contamination by common method bias. Notably, all variables in our study demonstrated VIF values below 3.3, suggesting that our measurement is free from common method bias, as shown in Table 5.

**Table 5:** VIF of latent variables.

	**FC**	**CL**	**FE**	**LB**	**PC**	**SA**
FC			1.000	1.335	1.000	1.000
CL	1.246					
FE				2.893		
LB						
PC				2.096		
SA				2.250		
TM	1.149					

### 4.4 Hypothesis testing

Hypothesis testing was conducted using SmartPLS 4.0 to construct a structural equation model. The analysis was conducted in two steps. In the first step, a serial mediation model was run without including any moderating effects. In the second step, the serial mediation model was modified by introducing trait mindfulness as a moderator. Both steps of the model analysis included control variables such as gender, age, grade, major, and daily WeChat usage time.

#### 4.4.1 Model fitting

To verify the model performance of the research model, alternative models 1 and 2 were constructed apart from the proposed model illustrated in Figure 1 (excluding moderating paths). Alternative model 1 omitted paths CL → OSA and FC → LB, transforming into a sequential mediation model, alternative model 2 removed the path FC → OSA, constituting a parallel mediation model. Upon examination (refer to Table 6), Stone-Geisser *Q*^2^-values are all >0 and exceed the threshold of 0.15 (Stone, 1974; Geisser, 1975), indicating sound predictive relevance.^2^ Other model fit indices, including Chi^2^, NFI, SRMR for three models are all within the recommended threshold range, endorsing a satisfactory model fit. Furthermore, compared to the proposed model, the Alternative model 1 and 2 exhibited lower values for Chi^2^, NFI, *Q*^2^, and increased SRMR values, indicating superior validity for the our proposed model.

**Table 6:** Model fit indices.

**Model**	**Chi^2^**	**SRMR (<0.08)**	**NFI (>0.90)**	***Q*^2^ (mean) (>0)**
Proposed model (Figure 1)	3,386.515	0.061	0.908	0.175
Alternative model 1 (sequential mediation)	3,483.782	0.067	0.903	0.171
Alternative model 2 (parallel mediation)	3,609.148	0.076	0.899	0.169

Consistent with SMARTPLS software's official guidance, the presented model fit indices in the table are intended for the estimated model, not the saturated one. Furthermore, in alignment with Stone (1974) and Geisser (1975), we apply the mean Q^2^-values of all endogenous variables to illustrate the predictive relevance of our structural equation model.

#### 4.4.2 Direct effect

**Figure 2:**
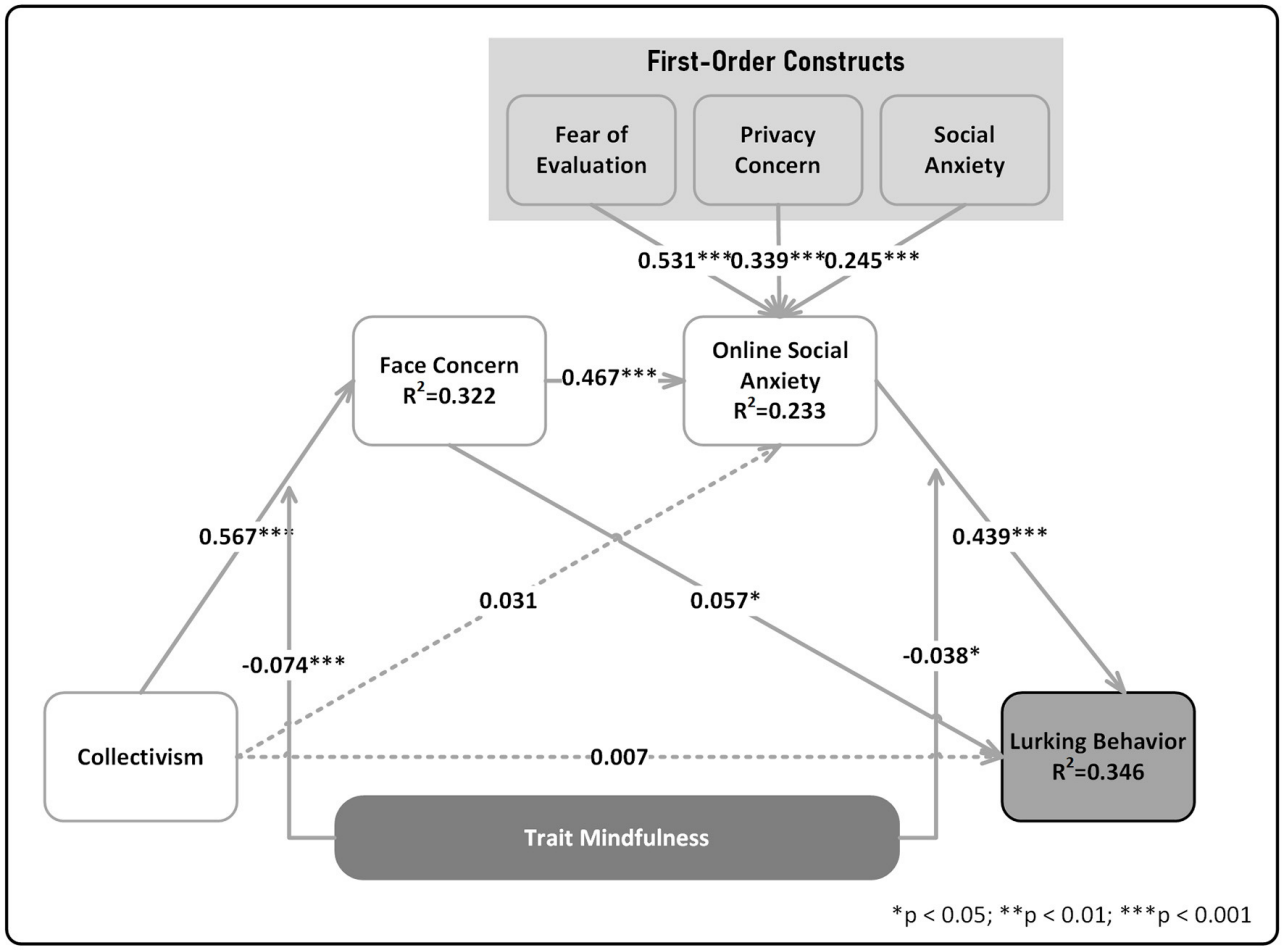
Result of structural model. The reported path coefficients and moderation effect coefficients in the figure represent an integration of the results from the two-step hypothesis testing conducted in this study, rather than a single model fit. *p < 0.05; **p < 0.01; ***p < 0.001.

Bootstrapping with resampling 5,000 times obtained path coefficients β and significance results of the relationship between variables, as shown in Table 7 and Figure 2.

**Table 7:** Direct effect path coefficients.

**Hypothesis**	**Path**	**β**	**LLCI**	**ULCI**	**β_0_**	**LLCI**	**ULCI**	**Findings**
H1	CL → FC	0.567^***^ (0.028)	0.513	0.622	0.544^***^ (0.028)	0.488	0.598	Supported
H2	CL → OSA	0.031 (0.037)	−0.038	0.107	0.045 (0.037)	−0.028	0.118	Not supported
H3	FC → OSA	0.467^***^ (0.034)	0.397	0.531	0.439^***^ (0.036)	0.367	0.507	Supported
H4	FC → LB	0.057^*^ (0.028)	0.002	0.112	0.059^*^ (0.029)	0.002	0.116	Supported
H5	OSA → LB	0.439^***^ (0.034)	0.373	0.505	0.444^***^ (0.034)	0.380	0.511	Supported
CL → LB	0.007 (0.033)	−0.054	0.072	0.017 (0.032)	−0.044	0.079	Not supported
Control variables	Gender	−0.092 (0.049)	−0.186	0.005				
	Age	0.007 (0.025)	−0.041	0.057				
	Grade	0.081^**^ (0.026)	0.030	0.131				
	Major	0.067 (0.051)	−0.035	0.163				
	Daily usage time	−0.059^**^ (0.022)	−0.104	−0.016				

In the table, β refers to the path coefficient of the model with moderator variables, β_0_ indicates the path coefficient of the model without moderator variables, and the standard errors are shown in parentheses.

^*^p < 0.05; ^**^p < 0.01; ^***^p < 0.001.

Figure 2 depicts that the *R*^2^ of key model variables FC, OSA, and LB were 0.322, 0.233, and 0.346, respectively. By the standards of Chin (1998) and Hansmann and Ringle (2004),^3^ the predictive power of the model had essentially reached a moderate level. By comparing β0 and β, no significant changes are seen in coefficient size, sign, or significance between models with and without moderator variables. This indicates that moderators didn't disturb model conclusions. According to the results of the β coefficients, Collectivism had a positive influence on face concern [β = 0.567, *p* < 0.001, 95% CI [0.513, 0.622]], supporting H1. However, the impact of collectivism on online social anxiety was positive but not significant [β = 0.031, *p* = 0.407, 95% CI [−0.038, 0.107]], failing to support H2. Face concern had a positive effect on online social anxiety [β = 0.467, *p* < 0.001, 95% CI [0.397, 0.531]], supporting H3. Face concern also had a positive influence on lurking behavior [β = 0.057, *p* < 0.05, 95% CI [0.002, 0.112]], supporting H4. Furthermore, online social anxiety had a positive effect on lurking behavior [β = 0.439, *p* < 0.001, 95% CI [0.373, 0.505]], supporting H5. Overall, the path analysis supported hypotheses H1, H3, H4, and H5.

#### 4.4.3 Mediation effect test

The mediation effects were examined using the Bootstrap method outlined by Zhao et al. (2010) and Hayes (2013). The results are presented in Table 8.

**Table 8:** Mediation effects test.

**Relationships**	**Effect**	**Boot SE**	**Boot LLCI**	**Boot ULCI**	***p***	**VAF**	**Hypothesis findings**	**Mediation type**
Total effect of CL → OSA	0.295^***^	0.035	0.230	0.365	0.000			
Total effect of FC → LB	0.262^***^	0.035	0.190	0.328	0.000			
Total effect of CL → LB	0.169^***^	0.033	0.104	0.237	0.000			
Direct effect of CL → LB	0.007	0.033	−0.054	0.072	0.831			
Total indirect effect of CL → LB	0.162^***^	0.027	0.112	0.217	0.000	95.86%		
**Specific indirect effect**								
CL → OSA → LB	0.013	0.016	−0.017	0.047	0.420	7.69%		
CL → FC → LB	0.033^*^	0.016	0.002	0.064	0.039	19.53%		
CL → FC → OSA	0.265^***^	0.024	0.217	0.314	0.000	89.83%	H6 supported	Full
FC → OSA → LB	0.205^***^	0.023	0.161	0.250	0.000	78.24%	H7 supported	Partial
CL → FC → OSA → LB	0.116^***^	0.015	0.088	0.147	0.000	68.64%	H8 supported	Partial

* *p* < 0.05; ^**^p < 0.01;

*** *p* < 0.001, VAF >80% indicated full mediation, 20%< VAF <80% showed partial mediation, VAF <20% illustrated no mediation (Hair et al., 2021). Owing to space constraints, results for the coefficients of the control variables were omitted from this table, which are the same as below.

In the mediation path of CL → FC → OSA, the total effect of collectivism (CL) on online social anxiety (OSA) was significant at 0.295 [*p* < 0.001, 95% CI [0.230, 0.365]]. The specific indirect effect was also significant at 0.265 [*p* < 0.001, 95% CI [0.217, 0.314]]. The variance accounted for (VAF) value was 89.83%, the results suggest full mediation, with face concern fully mediated the relationship between collectivism and online social anxiety, supporting H6.

In the mediation path of FC → OSA → LB, the total effect of face concern (FC) on lurking behavior (LB) was 0.262 [*p* < 0.001, 95% CI [0.190, 0.328]]. The specific indirect effect was significant at 0.205 [*p* < 0.001, 95% CI [0.161, 0.250]]. The variance accounted for (VAF) value was 78.24%, indicating nearly full mediation, which provided strong evidence for the mediating role of online social anxiety between face concern and lurking behavior, supporting H7.

In the serial mediation path CL → FC → OSA → LB, the total effect of CL → LB was significant at 0.169 [*p* < 0.001, 95% CI [0.104, 0.237]]. The specific indirect effect was 0.116 [*p* < 0.001, 95% CI [0.088, 0.147]], with a VAF value of 68.64%. These findings support the establishment of serial mediation between face concern and online social anxiety in the effect route of Collectivism-oriented lurking behavior (LB), supporting H8.

#### 4.4.4 Moderation effect test

Drawing upon hypotheses H9 and H10, this study incorporates interaction terms between trait mindfulness (TM) and CL as well as OSA in the moderation effect model to examine the moderated mediation paths of CL → FC and OSA → LB within the framework of PLS-SEM. The significance of the interaction term coefficients is used as the criterion for determining the presence of significant moderation effects. Table 9 showed that the interaction term of trait mindfulness and collectivism had a significantly negative influence on face concern [β = −0.047, *p* < 0.001, 95% CI [−0.103, −0.045]], demonstrating that trait mindfulness plays a negative moderating effect on the positive influence of collectivism on face concern, thereby supporting H9. The interaction term of trait mindfulness and online social anxiety also had a significant negative impact on lurking behavior [β = −0.038, *p* < 0.05, 95% CI [−0.071, −0.005]], indicating that trait mindfulness negatively moderated the path of online social anxiety to lurking behavior, H10 was supported.

**Table 9:** Moderation effects test.

**Moderated path**	**Relationships**	**β**	**SEs**	***t***	**Boot LLCI**	**Boot ULCI**	***p***	**Hypothesis**	**Findings**
CL → FC	CL → FC	0.524^***^	0.030	17.451	0.465	0.583	0.000	H9	Supported
	TM → FC	0.020	0.025	0.778	−0.028	0.071	0.436		
	TM × CL → FC	−0.074^***^	0.015	5.016	−0.103	−0.045	0.000		
	TM at−1SD	0.598^***^	0.028	21.715	0.544	0.651	0.000		
	TM at+1SD	0.450^***^	0.038	11.703	0.375	0.524	0.000		
OSA → LB	OSA → LB	0.395^***^	0.036	11.110	0.325	0.464	0.000	H10	Supported
	TM → LB	0.099^***^	0.028	3.564	0.046	0.154	0.000		
	TM × OSA → LB	−0.038^*^	0.017	2.261	−0.071	−0.005	0.024		
	TM at−1SD	0.433^***^	0.037	11.667	0.360	0.504	0.000		
	TM at+1SD	0.356^***^	0.041	8.623	0.275	0.437	0.000		

* *p* < 0.05; ^**^p < 0.01;

*** *p* < 0.001.

**Figure 3:**
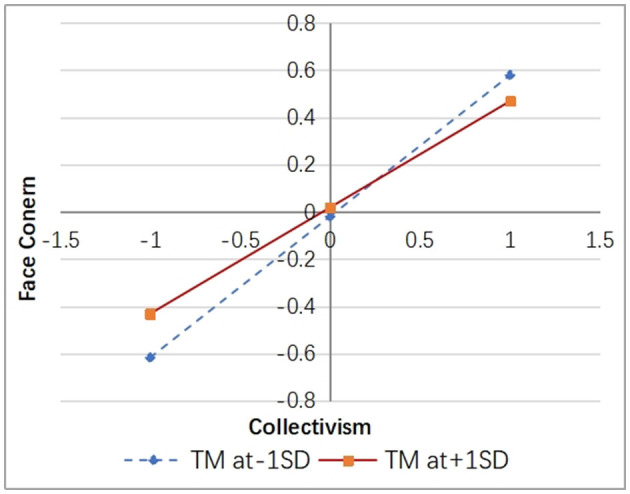
The moderating effect of trait mindfulness on collectivism and face concern.

**Figure 4:**
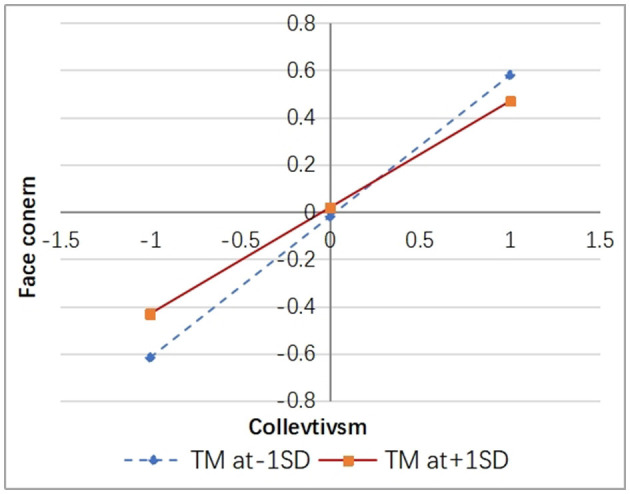
The moderating effect of trait mindfulness on online social anxiety and lurking behavior.

Simple slope analysis was conducted by adding and subtracting one standard deviation from the mean of TM, and the results were depicted in Figures 3, 4.

According to Figure 3, it is evident that university students with lower levels of trait mindfulness exhibit a stronger positive impact of collectivism on face concern [β = 0.598, *p* < 0.001, 95% CI [0.544, 0.651]]. Conversely, when individuals have higher levels of trait mindfulness, the positive influence of collectivism on face concern weakens [β = 0.450, *p* < 0.001, 95% CI [0.375, 0.524]], indicating that trait mindfulness can alleviate the reinforcing effect of collectivism on face concern.

According to Figure 4, it can be observed that university students with lower levels of trait mindfulness exhibit a stronger positive impact of online social anxiety on lurking behavior [β = 0.433, *p* < 0.001, 95% CI [0.360, 0.504]]. Conversely, individuals with higher levels of trait mindfulness demonstrate a relatively weaker positive effect of online social anxiety on lurking behavior [β = 0.357, *p* < 0.001, 95% CI [0.275, 0.437]], indicating that trait mindfulness can alleviate the reinforcing effect of online social anxiety on lurking behavior.

#### 4.4.5 Moderated mediation effect test

Moderated mediation analysis was employed to further validate the moderating effect of trait mindfulness. The PROCESS module in SmartPLS 4.0 was utilized for analysis, considering the implementation of formative Higher-Order latent variable structural equation model. Unlike SPSS's PROCESS plug-in, which can only deal with a single indicator, SmartPLS's PROCESS module automatically assigns equal weights to indicators in constructs with multiple indicators, aligning better with the requirements of our study. It should be noted that due to different algorithms, the path coefficients calculated based on PLS PROCESS are different from those based on the PLS algorithm. However, their direction and significance do not affect our judgment on moderated mediation and can provide robustness verification across algorithms. The moderated mediation effect was evaluated by testing the significance of the difference in Conditional Indirect Effects at plus and minus one standard deviation of the moderator trait mindfulness. Bootstrap with resampling 5,000 times was conducted, and the results are presented in Table 10.

**Table 10:** Moderated mediation effects test.

**Mediation path**	**Moderator (TM)**	**Conditional indirect effects**	**SEs**	**LLCI**	**ULCI**	**Difference between mean ±1SD**	**Differences' LLCI/ULCI**
Path1: CL → FC → OSA	at −1SD	0.342^***^	0.029	0.285	0.399	0.093	[0.013, 0.173]
	at Mean	0.296^***^	0.027	0.243	0.349		
	at +1 SD	0.249^***^	0.029	0.192	0.306		
Path2: FC → OSA → LB	at −1SD	0.230^***^	0.020	0.191	0.269	0.056	[0.001, 0.111]
	at Mean	0.201^***^	0.019	0.164	0.238		
	at +1SD	0.174^***^	0.020	0.135	0.213		
Path3: CL → FC → OSA → LB	at −1SD	0.151^***^	0.020	0.112	0.190	0.058	[0.008, 0.108]
	at Mean	0.121^*^	0.017	0.088	0.154		
	at +1SD	0.093^*^	0.016	0.062	0.124		

* *p* < 0.05; ^**^p < 0.01;

*** *p* < 0.001.

Table 10 reveals that the conditional indirect effects (Path1, Path2, and Path3) obtained from PLS PROCESS module are consistent with the specific indirect effects (path1 = 0.265, path2 = 0.205, path3 = 0.116) derived from the PLS algorithm in Table 8, which indicates the robustness of the study's findings.

The results of Path1 indicate that TM moderates the mediation pathway of CL → FC → OSA. The conditional indirect effect at mean-1SD [β = 0.342, 95%CI [0.285, 0.399]] and at mean+1SD [β = 0.249, 95%CI [0.192, 0.306]]. The difference in effect size between TM ± 1SD is 0.093, 95% CI [0.013, 0.173], that does not contain 0, indicating a significant moderated mediation effect.

The results of Path2 reveal that TM moderates the mediation pathway of FC → OSA → LB. The conditional indirect effect at mean-1SD [β = 0.230, 95% CI [0.191, 0.269]] and at mean +1SD [β = 0.174, 95% CI [0.135, 0.213]]. The difference in effect size between TM ± 1SD is 0.056, 95% CI [0.001, 0.111], that does not contain 0, demonstrating a significant moderated mediation effect.

The results of Path3 indicate that TM exerts a moderating effect on the serial mediation of CL → FC → OSA → LB. The conditional indirect effect at mean-1SD [β = 0.151, 95% CI [0.112, 0.190]] and at mean + 1SD [β = 0.093, 95% CI [0.062, 0.124]]. The difference in effect size between TM ± 1SD is 0.058, 95% CI [0.008, 0.108], that does not contain 0, which is evidence of a significant moderated serial mediation.

## 5 Discussion

### 5.1 Collectivism, face concern, and online social anxiety

The study revealed that collectivism has a positive impact on face concern, confirming H1. This finding aligns with Oyserman et al.'s (2002) study. Furthermore, it aligns with the socio-ecological view that collectivist societies (e.g., Japan and China) prioritize internal harmony and face-saving due to low relational mobility (Adams, 2005; Li et al., 2015, 2016). Therefore, a stronger sense of face is associated with a higher prominence of the collectivist syndrome, particularly among Chinese individuals (Oyserman et al., 2002). However, contrary to H2, collectivism does not have positive effect on online social anxiety, which differs from the findings of Agishtein and Brumbaugh (2013) and Frías et al. (2013). Instead, our results provide further support for the evolutionary perspective that collectivism serves as an adaptive “anti-psychiatry” mechanism (Wu et al., 2018). By promoting social connections and establishing social norms, collectivism mitigates the risks associated with personality exposure, leading to lower rates of anxiety and depression among individuals in collectivist societies (Kessler and Üstün, 2008). Interestingly, the confirmation of H3 demonstrates the significant positive association between face concern and online social anxiety, in line with the findings of Liu and Cheng (2023). Specifically, a higher level of face concern corresponds to a greater manifestation of online social anxiety. Drawing on Social Exchange Theory, face concern serves as a symbolic resource for interpersonal relationships and social status in China (Hwang, 1987). Under the influence of this factor, Chinese university students, driven by their face concern, exhibit a heightened sense of vigilance regarding their verbal and behavioral expressions on their WeChat Moments, which subsequently contributes to elevated levels of social anxiety among this population (Liang and Duan, 2018). Additionally, the confirmation of H6 demonstrates that collectivism and face concern jointly contribute to the sources of pressure leading to online social anxiety among Chinese university students. Furthermore, as a primary channel and amplifier of collectivism, face concern exacerbates the levels of online social anxiety.

### 5.2 Face concern, online social anxiety, and lurking behavior

According to the ISTO (Individual, Social, Organizational, and Technical) paradigm, lurking behavior is influenced by individual, technical, societal, and organizational factors, such as social interaction and trust within online communities (Nguyen, 2021). However, these factors have not been sufficiently addressed in prior studies. Therefore, for the first time, we incorporated face concern with Chinese social traits as an key variable in the study of “Chinese-style lurking.” The study findings provides empirical support for H4, revealing a positive influence between face concern and lurking behavior among Chinese university students. Specifically, a stronger degree of face concern is associated with a more pronounced manifestation of Wechat lurking. Diverging from previous research (Nguyen, 2021; Juan et al., 2023), this finding contributes to the existing literature by expanding the range of explanatory variables and enhancing our understanding of lurking behavior in the digital context. Furthermore, consistent with H5, our study reveals a significant positive relationship between online social anxiety and lurking behavior. According to the cognitive-behavioral model of social anxiety, Chinese university students engage in lurking behavior on social media platforms due to the anxiety they perceive within their WeChat Moments. This passive usage behavior or maladaptive coping strategy further diminishes their intention to continue using Wechat, leading to a cumulative tendency toward lurking (Weidman and Levinson, 2015), which explains the increasing prevalence of lurking in recent years (Ortiz et al., 2018) and the persistently high proportion of lurkers (ranging from 45.5 to 90%; Fullwood et al., 2019).

Notably, online social anxiety and lurking behavior show remarkable similarity in symptoms between Chinese and Western studies. However, it is not enough to assume that their underlying mechanisms are identical solely based on the similarity of symptoms. In the context of Chinese culture, online social anxiety and lurking behavior are influenced by not only the concerns related to saving and earning face (Belk, 1988; Bao et al., 2003), but also by the mediating role of online social anxiety in the relationship between face concern and social media lurking, as proved in our H7. Moreover, drawing upon the seminal model of person-culture fit hypothesis in psychology (Oishi et al., 1999), it is crucial to ground our analysis within the cultural framework of collectivism and consider the phenomenon of “Chinese-style lurking” that extends beyond the symptom pool typically associated with it. Furthermore, our research has provided evidence supporting H8: that face concern and online social anxiety play a serial mediating role between collectivism and lurking behavior. This implies that the self-concept of Chinese collectivist values, influenced by the traditional significance of face concern, jointly shapes the pressures of social psychology and behavior among contemporary Chinese university students. These pressures manifest in individuals' excessive fear of potential embarrassment or humiliation experienced by others or the collective they represent. Consequently, this adaptive response to anxiety triggers the occurrence of lurking outcome.

### 5.3 Negative moderating effects of trait mindfulness

Face is fundamentally a socio-self construction issue, as Ting-Toomey and Kurogi (1998) pointed out. Chinese's face concern is more closely tied to collectivist cultural values compared to that in Western society, although the concept of face is not limited to a specific culture. In the context of China's collectivist traditions, the psychological need for affiliation and belongingness (Gambrel and Cianci, 2003) render collectivists highly sensitive to how their behavior and actions are perceived and evaluated by others (Ndubisi and Moi, 2005). Consequently, this sensitivity becomes an external stress, inadvertently leading to a detachment from individual self-awareness and self-esteem experiences. According to the Mindfulness Stress Buffer Theory, mindfulness has been found to effectively mitigate stress appraisal and response. As corroborated by H9 in this study, trait mindfulness serves as a negative moderator in the relationship between collectivism and face concern.

Previous research demonstrates the effectiveness of mindfulness in promoting self-awareness (Chen et al., 2019) and increasing self-esteem levels (Dekeyser et al., 2008; Gámez-Guadix and Calvete, 2016), thus effectively regulating the cultivation of face concern. Supporting H10, this study demonstrates that trait mindfulness negatively moderates the association between online social anxiety and lurking behavior. Specifically, Chinese university students' lurking behavior is primarily driven by online social anxiety, which stems from negative fantasies about potential future distresses or fear-inducing situations that have not yet occurred, as well as the perception of subjective inertial experience. Which is not focused on present self-presentation and experiences. Trait mindfulness refers to individual's ability to concentrate on the present moment (Gu et al., 2022). By fostering cognitive and behavioral flexibility, trait mindfulness facilitates adaptive responses to present situations, reducing reliance on habitual or impulsive reactions (Baer, 2003; Bishop et al., 2004). By exerting its influence, trait mindfulness effectively enhances cognitive and behavioral flexibility, enabling individuals to respond adaptively to present situations rather than relying on habitual or impulsive reaction (Baer, 2003; Bishop et al., 2004). As a result, trait mindfulness can effectively decrease levels of online social anxiety, leading to an improvement in the behavior associated with social media lurking.

## 6 Implication

This study investigates the causes and underlying mechanisms of social media anxiety and lurking behavior among Chinese university students, considering the influence of collectivism and face concern, as well as the role of trait mindfulness. The results reveal that collectivism has a positive influence on online social anxiety via face concern, which in turn fosters lurking behavior. Face concern and online social anxiety play a serial-mediation role between collectivism and lurking behavior, trait mindfulness acts as a negative moderator in the pathway of collectivism-induced face concern and online social anxiety-driven lurking behavior. In terms of marginal contribution, Our study brings a theoretical advancement by introducing an integrated model of collectivism, face concern, online social anxiety, and lurking behavior, providing a comprehensive understanding of the underlying mechanisms behind the distinct behavior of “Chinese-style lurking” while expanding the scope of research on lurking behavior. Furthermore, this study extends the application of the mindfulness stress-buffering theory as a novel moderating factor, broadening its interdisciplinary relevance to the fields of health communication and media studies.

In practical terms, firstly, it is imperative for social media platforms to recognize the significant role of a positive community culture in transforming users' perceptions, emotional needs, and information sharing. To effectively mitigate the trend of social media lurking, platforms should strive to create a supportive environment while minimizing malicious feedback and dissemination. This will contribute to an enhanced level of comprehensive governance on social media platforms. Secondly, unlike other traits, trait mindfulness is malleable and can be effectively enhanced through interventions such as mindfulness training (Khoury et al., 2015). For individuals with severe online social anxiety disorder, targeted mindfulness training programs led by experts, opinion leaders, or social organizations can reduce stress, alleviate anxiety symptoms, and decrease individuals' tendency to engage in social media lurking or silence. Lastly, it is highly recommended for universities, organizations, and media platforms to proactively cultivate a positive and inclusive online social environment among university students. This approach will effectively mitigate the adverse outcomes associated with excessive lurking, such as social isolation and apathy, while concurrently promoting active engagement and meaningful participation in broader societal discourse.

## 7 Limitations and prospects

This study has several limitations. Firstly, the research data was collected through convenient online sampling, and the use of self-administered questionnaires may not entirely eliminate common method bias. Secondly, the research model may overlook the influence of other potential control variables on the research outcomes. For instance, the quantity and quality of a user's social media connections can also impact their level of evaluation anxiety and the cost associated with social media lurking. Future investigations should consider including additional control variables to enhance the precision of the research findings. Thirdly, broadening discussions and deepening research that holds relevance to cultures or backgrounds outside the traditional focus on East Asia and the West carries significant potential research value. For instance, Latin American societies, much like their East Asian counterparts deeply influenced by Confucianism, place a strong emphasis on collectivist values, while also giving weight to various forms of individual self-expression often associated with Western societies (Krys et al., 2022). This broadens the interpretive scope for exploring the link between collectivism and social media lurking behavior. When different cultural backgrounds are intertwined with these specific research contexts, our conclusions may veer toward complexity and possibly counterintuitive results. For example, evidence suggests that the level of individualism in the East is often underestimated and the West is not as overwhelmingly individualistic as frequently perceived. These concepts are inherently more intricate than conventionally acknowledged (Lomas et al., 2023). Regardless, these insights serve to challenge and expand upon the existing cross-cultural paradigms within this field, making way for future research that is more nuanced and profound. Fourthly, this paper merely proposes a possible pathway for the impact of Chinese-style lurking behaviors, the complexity of which necessitates further exploration. Potential areas of in-depth inquiry might include the concrete influence mechanisms of collectivism on face-saving awareness, facework in cross-cultural studies, emerging forms of anxiety under the usage scenarios of Chinese social media, the impact and governance mechanisms of Mindfulness-Based Stress Reduction (MBSR) in online social anxiety, as well as the dynamics of lurking behaviors across various social media platforms and cultural contexts. Moreover, the long-term effects of lurking behaviors on individual societal and psychological health may also serve as a fruitful domain for future research.

## 8 Conclusion

This study uncovers the unique causes and mechanisms of Chinese-style lurking among university students in the context of collectivism and face concern, and validates the moderating role of trait mindfulness. Findings suggest that collectivism positively affects face concern, which in turn affects online social anxiety. Face concern acts as a full mediator between collectivism and online social anxiety, establishing a serial mediated pathway between face concern, online social anxiety, and lurking behavior in the context of collectivism. Trait mindfulness negatively moderates the paths from collectivism to face concern and from online social anxiety to lurking behavior, creating a moderated serial mediation. Our study expands the research on online lurking behavior by integrating culturally-specific factors in China, presenting a unique investigation into the moderating effect of mindfulness on Chinese-style lurking behavior. The findings provide insights for addressing pervasive issues such as silence, isolation, indifference, and asocial tendencies that arise from extensive lurking on social media platforms, and propose strategies to enhance youth engagement, opinion expression, and social participation.

## Data availability statement

The original contributions presented in the study are included in the article/Supplementary material, further inquiries can be directed to the corresponding author.

## Ethics statement

This study was reviewed and approved by the Academic Committee of the School of Journalism and Communication of Huaqiao University. Written informed consent from the patients/participants was not required to participate in this study in accordance with the national legislation and the institutional requirements.

## Author contributions

BH: Writing – original draft, Writing – review & editing. YZ: Writing – original draft, Writing – review & editing. CL: Writing – original draft, Writing – review & editing. SZ: Investigation, Writing – original draft. ZZ: Investigation, Writing – original draft. RB: Investigation, Writing – original draft.

## Footnotes

^1^Jarvis et al. (2003) proposed two important criteria for determining formative constructs: Firstly, the assumed causal direction from items to constructs poses is problematic. Secondly, removing an indicator may change the conceptual meaning of the construct. Evidently, online social anxiety logically originates from pre-existing social anxiety rather than the other way around. Additionally, if the first-order construct “social anxiety” is removed from the measurement of online social anxiety, its definition change significantly. Accordingly, online social anxiety is more appropriately defined as a formative second-order construct.

^2^According to Cohen's (1988) study, *Q*^2^-value greater than zero indicates acceptable predictive relevance. Further, values of 0.02, 0.15, and 0.35 signify that an exogenous construct has small, medium, or large predictive relevance, respectively.

^3^According to Chin (1998) and Hansmann and Ringle (2004), *R*^2^-values of ~0.670 are considered substantial, values around 0.333 moderate, and values around 0.190 weak.
